# Management of Portal Vein Thrombosis in a Postpartum Patient Using Combined Endovascular Therapy and Anticoagulation

**DOI:** 10.7759/cureus.101109

**Published:** 2026-01-08

**Authors:** Shannon Chu, Fahad Farooq, Zohaib Khan, Tomas Mujo

**Affiliations:** 1 Radiology, State University of New York Upstate Medical University, Syracuse, USA

**Keywords:** catheter-directed thrombolysis, endovascular therapy, interventional radiology, mechanical thrombectomy, portal vein thrombosis, postpartum complications, postpartum endometritis, retained products of conception, superior mesenteric vein thrombosis, venous thromboembolism

## Abstract

Portal vein thrombosis (PVT) is a rare but potentially life-threatening condition in the postpartum period. We present the case of a 25-year-old female who developed extensive thrombosis involving the portal vein, superior mesenteric vein, and splenic vein 13 days after an at-home vaginal delivery complicated by manual placenta extraction performed at a hospital. She presented with progressive abdominal pain and was found to have postpartum endometritis, retained products of conception, and ascites. Laboratory evaluation revealed leukocytosis and mild hepatic enzyme elevation, with a largely negative thrombophilia workup. Given the extensive thrombosis involving the portal vein, superior mesenteric vein, inferior mesenteric vein, and splenic vein, accompanied by ascites and multiple risk factors, she underwent urgent catheter-directed thrombolysis and mechanical thrombectomy in addition to systemic anticoagulation. Post-procedural imaging confirmed significant restoration of portal venous flow. The patient was transitioned to oral anticoagulation and discharged in stable condition. This case highlights the importance of early recognition and multidisciplinary management of postpartum PVT. In patients with extensive clot burden or risk factors for failed recanalization, endovascular therapy combined with anticoagulation may offer a superior outcome to anticoagulation alone.

## Introduction

Pregnancy is a physiological hypercoagulable state that puts women at risk for venous thromboembolism (VTE) during the antepartum and postpartum period [1,2]. Most venous thromboembolism cases are observed in the deep veins of the lower extremities or the lungs [1]. About 80% of thromboembolic events occurring after childbirth take place within the first three weeks [2,3]. The elevated risk continues for up to 25 weeks postpartum [4].

Portal vein thrombosis (PVT) is a rare occurrence in the antepartum and postpartum period [5]. If left undetected, a PVT may progress to involve more proximal mesenteric veins, such as the superior mesenteric vein, leading to a higher risk of intestinal necrosis and mortality [6,7].

In this case report, we aim to report a progressive extension of a PVT in a patient with postpartum endometritis and retained products of conception, treated medically and with interventional therapy before making a successful recovery.

## Case presentation

A 25-year-old female G3P2012 with a history of obesity, gastric bypass surgery, migraines, and anxiety/depression, presented to the hospital 13 days postpartum of a 36-week gestation home delivery that required a manual placental extraction at a hospital. Since delivery, she experienced worsening generalized abdominal pain, most prominent in the epigastric and right lower abdominal quadrant, leading her to seek emergency care.

**Figure 1:**
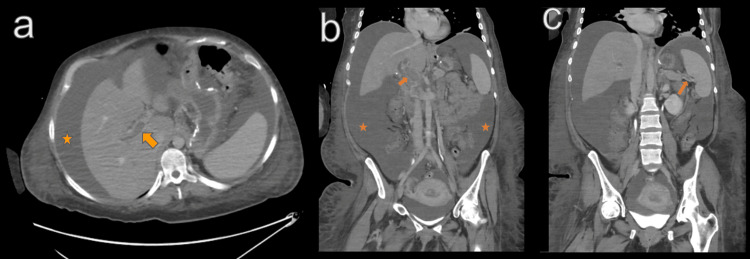
Axial (a) and coronal (b, c) contrast-enhanced CT of the abdomen demonstrating extensive portal vein and splenic vein thrombosis (arrows) and extensive abdominal ascites (star).

**Figure 2:**
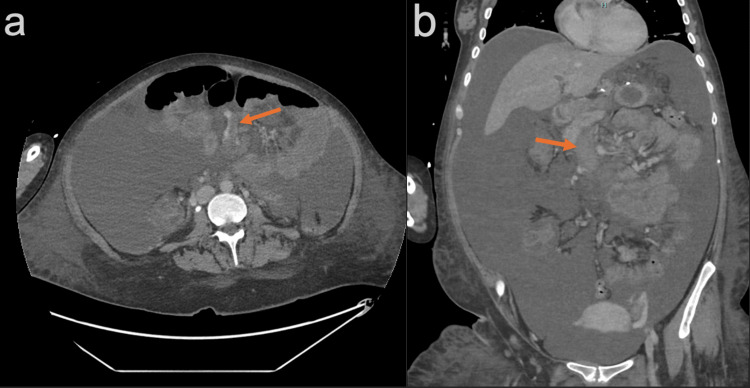
Axial (a) and coronal (b) contrast-enhanced CT of the abdomen demonstrating occlusion of the superior mesenteric vein (arrows).

She was transferred from an outside hospital to our institution with suspicion of retained products of conception. Relevant laboratory values were obtained at the time of admission (Table 1). Of note, her international normalized ratio (INR) was elevated at 1.75, likely due to liver dysfunction, which was also suggested by the presence of hypoalbuminemia. External imaging records included a transabdominal pelvic ultrasound and a contrast enhanced CT scan of the abdomen and pelvis; findings showed a post gravid uterus with an endometrial heterogenous complex, a large amount of free fluid in the pelvis and adnexa, acute hemorrhagic change within the endometrial canal, and evidence of a new thrombus with near complete occlusion of the portal venous system, splenic vein, superior mesenteric vein (SMV), and inferior mesenteric vein (IMV) (Figures 1-2).

**Table 1:** Relevant laboratory values at admission. WBC: white blood count; ALT: alanine aminotransferase; AST: aspartate aminotransferase; INR: International normalized ratio; µL: microliter; g: gram; dL: deciliter; mmol: millimole per liter; U: units; L: liter; s: seconds; dRVVT: dilute Russell’s viper venom time

Test	Result	Reference Range	Units
WBC	31,400	4,000 – 10,000	/µL
Hemoglobin	9.8	11.5 – 15.5	g/dL
Hematocrit	30.8	36 – 45	%
Platelet count	520,000	150,000 – 400,000	/µL
Lactate	1.1	0.5 – 2.2	mmol/L
Alkaline phosphatase	220	44 – 147	U/L
ALT	22	< 33	U/L
AST	26	< 32	U/L
Albumin	1.7	3.5 - 5.2	g/dL
Prothrombin time	20.2	11.6 – 14.0	s
INR	1.75	< 1.1	-
Cardiolipin IgG antibody	3.7	< 20	U/mL
Cardiolipin IgM antibody	1.7	< 20	U/mL
Beta-2 glycoprotein IgG antibody	< 6.4	< 20	U/mL
Beta-2 glycoprotein IgM antibody	< 1.1	< 20	U/mL
Lupus anticoagulant screen (dRVVT ratio)	0.91	< 1.2	-
Hexagonal phase phospholipid neutralization assay	11.5	< 8	s
JAK2 kinase molecular testing	Negative	Negative	-

She was diagnosed with extensive PVT, postpartum endometritis, and retained products of conception. Medicine, obstetrics and gynecology (OBGYN), vascular surgery, interventional radiology (IR), and hematology were consulted. Vascular surgery was initially consulted and recommended continuation of the heparin drip that had been initiated in the emergency department. Of note, the patient was not breastfeeding and was not actively bleeding. OBGYN treated the patient’s retained products of conception and postpartum endometritis with a suction dilation and curettage and IV gentamicin and ampicillin, respectively. Due to concerns of bacterial peritonitis, her antibiotics were changed to ceftriaxone and metronidazole. She underwent a diagnostic and therapeutic paracentesis, which drained 6 L of cloudy, serous fluid, and was given albumin 25 g.

Her abdominal exam worsened; thus, the vascular team recommended referral to IR. General surgery was also consulted for concerns of bowel ischemia. Due to extensive portal and mesenteric venous thrombosis compounded by large-volume ascites, suspected bacterial peritonitis, and significant prothrombotic risk factors, endovascular intervention was indicated, and the patient was urgently taken to the IR suite. Under ultrasound guidance, 1500 cc of serous ascitic fluid was drained from the right lower quadrant using a 6 French (Fr) locking Skater pigtail catheter. The decision to drain ascites first was multifactorial: (1) drainage improves procedural visualization and facilitates safe transhepatic access; (2) removal of ascites may reduce portal venous pressure; and (3) in the event of hemorrhagic complications, bleeding control is more challenging in the presence of ascites.

Right common femoral arterial access was obtained using the modified Seldinger technique. A 5 Fr sheath was placed, and a Cobra C2 catheter was advanced into the superior mesenteric artery (SMA). SMA angiography demonstrated delayed and faint opacification of the portal venous system, consistent with extensive thrombosis. A total of 9000 units of heparin was administered via the catheter for standard procedural anticoagulation during SMA angiography to reduce the risk of catheter and wire-induced thrombosis, and to prevent thrombus propagation in the setting of severely impaired mesenteric and portal venous flow. Here, the intent of intraprocedural heparin administration was procedural prophylaxis rather than treatment of the underlying portal venous thrombosis.

**Figure 3:**
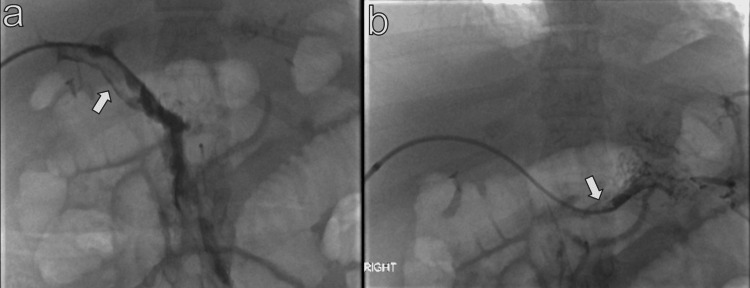
Fluoroscopic spot image of the contrast instilled portal vein (a) and splenic vein (b) demonstrating filling defects identified as thrombus (arrows).

A percutaneous transhepatic approach was selected because it provides the most direct and stable access to the superior mesenteric and splenic venous systems, which was necessary given the extensive thrombus burden involving the SMV, IMV, splenic vein, and main portal vein. The primary goal was rapid thrombus removal and restoration of mesenteric venous outflow. Additionally, large-bore aspiration thrombectomy devices required for effective clot debulking are more readily accommodated via transhepatic access. Transhepatic access to a right portal vein branch was established under ultrasound and fluoroscopic guidance using a 21-gauge Cook Chiba needle. Over an Amplatz wire, a 9 Fr 23 cm vascular sheath was advanced into the main portal vein. Portography revealed extensive thrombus involving the right, left, and main portal veins, as well as the SMV, IMV, and splenic vein (Figure 3). Using a 5 Fr Angiodynamics Soft-Vu Kumpe catheter (Angiodynamics, Latham, New York, USA) and a 0.035" Terumo Angled Glidewire (Terumo Medical Corporations, Somerset, New Jersey), the SMV was selected, and venography confirmed extensive thrombus.

**Figure 4:**
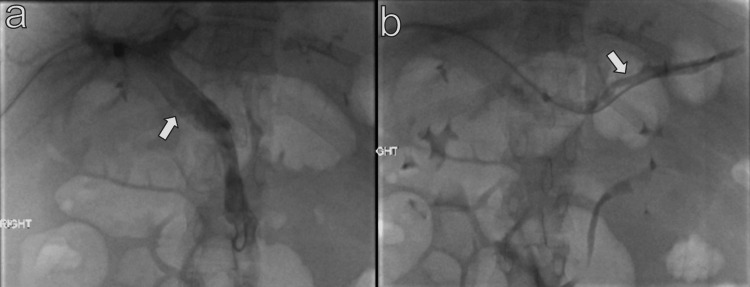
Fluoroscopic spot image of the contrast instilled portal vein (a) and splenic vein (b) post-embolization, demonstrating significant resolution of the filling defects identified as thrombus (arrows).

**Figure 5:**
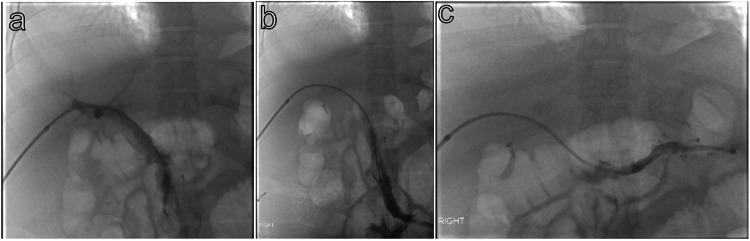
Post-aspiration thrombectomy portal venogram shows improved opacification of the main portal vein (a), superior mesenteric vein (b), and splenic vein (c).

Mechanical thrombectomy was performed using a 7 Fr Lightning 7 system (Penumbra, Inc., Alameda, California, USA), which was inserted through the 9 Fr vascular sheet across the SMV, IMV, splenic vein, and portal vein branches. A 10 mg tissue plasminogen activator (tPA) bolus was administered via the 7 Fr sheath, followed by aspiration thrombectomy. Post-intervention venography demonstrated substantial improvement in portal flow with a minimal residual filling defect at the portal confluence (Figures 4-5). A 5 Fr Angiodynamics Omni Flush pigtail catheter (Angiodynamics, Latham, New York, USA) was positioned in the SMV for ongoing catheter-directed thrombolysis (tPA at 1 mg/h) and concurrent heparin infusion (60 units/h). Hemostasis was achieved of the common femoral artery using manual pressure, an Angio-Seal closure device (Terumo Medical Corporation, Somerset, New Jersey), and skin closure cyanoacrylate glue (Dermabond; Ethicon, Somerville, New Jersey). The patient was transferred to the intensive care unit for post-procedural care.

The following day, the patient returned to the IR suite for lysis catheter reassessment. Portal venography via the Omni Flush catheter demonstrated improved flow throughout the portal venous system. Portal pressure measured 8 mmHg, consistent with mild portal hypertension. The catheter was exchanged for a Kumpe catheter to evaluate the splenic vein, which was patent. After confirming adequate thrombus resolution, the catheter and sheath were withdrawn, and the transhepatic tract was embolized to the liver capsule using detachable and pushable coils. Final hemostasis was achieved with manual compression and skin closure with cyanoacrylate glue. The procedure was technically successful with marked improvement in portal venous flow.

Following the IR procedure, the patient’s abdominal pain improved, and she had a series of benign abdominal exams; however, she had persisting moderate ascites. Her ascitic fluid culture results suggested *Streptococcus*/*Enterococcus*, and she was treated with a total 10-day course of ceftriaxone and metronidazole. Her ascites improved with IV furosemide 40 mg twice a day, and later transitioned to oral furosemide 40 mg twice a day. Prior to discharge, she was transitioned from heparin drip to apixaban 10 mg twice a day for seven days, with a plan to continue oral anticoagulation medication for one year under the care of a hematologist. Her INR stabilized in the 1.4-1.5 g/dL range.

## Discussion

An acute PVT is characterized by the rapid development of a clot in the portal vein [8]. It can extend to portions of the mesenteric veins and/or the splenic vein. Acute PVT may clinically present with either sudden onset or gradually progressing abdominal pain over a few days [8]. The range and severity of symptoms vary depending on the degree of obstruction, extension of the thrombus into nearby vasculature, inflammation, and presence of comorbidities such as sepsis. The diagnosis of an acute PVT can be rapidly established using Doppler sonography, showing the absence or decrease of flow [8]. CT or MR angiography is a more sensitive method than Doppler sonography for evaluating thrombus extension [8].

The standard therapy for acute PVT unrelated to cirrhosis or malignancy is to start with a low molecular weight heparin to achieve rapid systemic anticoagulation and continue long-term anticoagulation therapy to prevent recurrence [7-10]. The European Network for Vascular Disorders of the Liver (EN-Vie) prospective multicenter study (n=102) concluded that early anticoagulation alone was effective to obtain recanalization of acute PVT in one-third of patients [9]. However, early anticoagulation is ineffective at achieving PVT recanalization when there is ascites, an extensive thrombus, splenic vein involvement, and/or two or more prothrombotic factors [9-11]. Currently, there are no established interventional treatment guidelines for acute PVT in noncirrhotic patients. The goal of endovascular therapy is to remove the thrombus, restore outflow, and prevent thrombus extension and permanent sequelae. Catheter-directed thrombolysis (CDT), mechanical thrombectomy, and transjugular intrahepatic portosystemic shunt (TIPS) are types of endovascular therapy approaches that have high success rates of recanalization but may result in adverse events such as major bleeding [10,12]. Recent research proposes that endovascular therapy for acute PVT in noncirrhotic patients should be considered when systemic anticoagulation fails and/or if there are signs of bowel ischemia [10,12].

Acute PVT is a rare occurrence in the postpartum period. Other reported cases of postpartum PVT were in the setting of cesarean sections, an underlying antiphospholipid syndrome, hematologic disorder, and/or abdominal trauma [6, 13-15]. In those reported cases, patients experienced good recovery with standard anticoagulation therapy. In this case study, our patient underwent an at-home vaginal delivery and had a negative thrombophilia workup. She presented with a complete to near-complete thrombotic obstruction of the portal venous system, splenic vein, and SMV in the setting of postpartum endometritis and retained products of conception. For our patient, anticoagulation alone would not be effective at obtaining recanalization since she had bacterial peritonitis, ascites, and an extensive thrombus with splenic vein involvement. Moreover, having sepsis from postpartum endometritis, bacterial peritonitis, and retained products of conception placed her at risk for disseminated intravascular coagulation. Due to the severity of her case, it was important to mitigate the risk of further thrombosis and intestinal ischemia sequela. Based on the existing evidence, adding endovascular therapy to standard treatment may be advantageous for acute PVT with large blood clots, ascites, occluded splenic veins, and two or more prothrombotic factors. The patient was treated with IR CDT and mechanical thrombectomy in addition to anticoagulation therapy due to concerns for an incomplete recanalization on anticoagulation therapy alone and an elevated risk of mortality from comorbidities.

## Conclusions

In this case report, we presented a 13-day postpartum patient with worsening abdominal pain, ultimately diagnosed with extensive PVT, ascites, postpartum endometritis, and retained products of conception. This case highlights the potential benefit of combined endovascular therapy in managing acute portal venous thrombosis in a postpartum patient with a large clot burden, multiple comorbidities, and a life-threatening condition.
